# Analyzing Particularities of Sensor Datasets for Supporting Data Understanding and Preparation

**DOI:** 10.3390/s21186063

**Published:** 2021-09-10

**Authors:** Francisco Javier Nieto, Unai Aguilera, Diego López-de-Ipiña

**Affiliations:** 1 Research and Innovation, ATOS Spain SA, 48013 Bilbao, Spain; 2 DeustoTech, University of Deusto, 48007 Bilbao, Spain; unai.aguilera@deusto.es (U.A.); dipina@deusto.es (D.L.-d.-I.)

**Keywords:** sensor data analytics, internet of things, data understanding, anomaly detection, data analysis parallelization

## Abstract

Data scientists spend much time with data cleaning tasks, and this is especially important when dealing with data gathered from sensors, as finding failures is not unusual (there is an abundance of research on anomaly detection in sensor data). This work analyzes several aspects of the data generated by different sensor types to understand particularities in the data, linking them with existing data mining methodologies. Using data from different sources, this work analyzes how the type of sensor used and its measurement units have an important impact in basic statistics such as variance and mean, because of the statistical distributions of the datasets. The work also analyzes the behavior of outliers, how to detect them, and how they affect the equivalence of sensors, as equivalence is used in many solutions for identifying anomalies. Based on the previous results, the article presents guidance on how to deal with data coming from sensors, in order to understand the characteristics of sensor datasets, and proposes a parallelized implementation. Finally, the article shows that the proposed decision-making processes work well with a new type of sensor and that parallelizing with several cores enables calculations to be executed up to four times faster.

## 1. Introduction

Several works, such as refs. [1,2], have already reported that sensor data is not perfect and datasets usually include erroneous information. They identify two main problems: (a) missed readings (that are not transmitted for some communication reason) and (b) unreliable readings (faulty values reported by the sensors). Therefore, it is necessary to carry out a data cleansing procedure, so it will be possible to (i) avoid or minimize data leakage when working in machine learning (ML) models and (ii) analyze and use the data appropriately in Big Data pipelines.

The CrowdFlower Data Science report states that data scientists spend 51% of their time “collecting, labeling, cleaning and organizing data” [3]. The Kaggle survey for data scientists [4] was more specific, finding that 15% of their time (on average, with a maximum of 24%) was only for cleaning data. Anaconda conducted a survey in 2020 [5], reporting that data scientists spend 26% of their time in data cleansing.

Such activity deals with the identification of missing values (and filling in gaps), identifying outliers (and removing them), dealing with noise and avoiding duplication. As these tasks are time-consuming, an important part of research has been focused on the automation of these activities.

On the other hand, similar techniques are used to analyze sensor behavior, so it will be possible to identify anomalies and study the “quality” of a sensor and the data it produces. This is particularly useful in platforms that manage a large set of sensors and need to monitor if they are working properly, thus making it possible to determine whether the applications can trust the information they provide.

In order to apply all of these techniques, it is necessary to understand the data in better detail so researchers and practitioners can adapt their methods. We believe that, sometimes, certain assumptions about datasets are made but are not always true (such as the normality of the data), and thus require formal analysis. Therefore, our hypothesis is that an adequate analysis based on statistics allows an understanding of the basic and hidden characteristics of data provided by sensors from multiple domains. We propose that such analysis will be crucial in later stages and, although some early analysis of the data might have a rather high computational cost (as in edge environments), exploiting the parallelization provided by current technologies makes this data pre-processing feasible.

This work sought to understand the datasets generated by several types of sensors (used in several domains, such as smart cities and agriculture), identifying potential issues and particularities that, when cleaning and analyzing the data, need to be addressed and considered. We believe that there are two main topics that are related to the quality of the data: how the data vary and the presence of outliers. Therefore, we identified several research questions that require solutions through experimentation:

- *RQ1: Which aspects of sensors are linked to the variation of data and the potential presence and role of outliers? Are we making some assumptions that are not met?*
- *RQ2: Which statistical solutions can provide valuable information about these aspects, so that we can analyze them and understand how they behave for different sensors? How can we use, combine and interpret their outcomes?*
- *RQ3: In case outliers are present, how do they affect the basic characteristics of sensors’ data? Are there alternatives that could mitigate the problem?*

Additionally, because correlation is a method that can be used to compare the behavior of similar sensors and extract additional information linked to the context of sensors, we defined additional research questions:

- *RQ4: How does correlation work with different types of sensors? What is the potential utility of correlation in understanding data?*
- *RQ5: How do the different solutions perform? Are they also affected by outliers? How?*

As a result, we wanted to propose a solution that could facilitate all of this analysis for different datasets:

- *RQ6: Is it possible to define a set of processes, as a way to automate and formalize data understanding, applicable to different domains?*
- *RQ7: Can we exploit computational resources in a better way, increasing the efficiency of the proposed solution?*

We acknowledge that aspects such as data buffering, synchronization and the management of missing values are important (as analyzed in some works [6,7]), but they require specific analyses and are out of the scope of this work (although the results could be useful to support solutions in some of those topics). We assume that such aspects are managed at the data collection level, such that the data received contain measurements at regular intervals and that missing values are indicated with some specific number (e.g., −99.9 in our case).

The paper is organized as follows. Section 2 reviews previous work on data cleaning and the detection of anomalies in data coming from sensors. Section 3 describes the process followed and data sources used to perform an analysis of data cleansing issues. Section 4 describes the results of the observations in sensor data pre-processing and presents the proposed processes for supporting data understanding, together with the results of applying such processes to a type of sensor not used before. Finally, Section 5 presents the discussion and conclusions, including future work.

## 2. Related Work

In recent years, there have been several works analyzing how to clean data from different sources (many of them focused on climate data). Works such as refs. [7,8] discuss how to fill gaps in weather data and the usage of certain tests (such as the standard normal homogeneity test [9], Pettitt test [10], Buishand range test [11], etc.) to determine outliers in the data.

Some works have analyzed the types of errors produced by sensors when collecting data. This is the case in refs. [12,13], which identified a similar classification (random/malfunction, bias and drift/noise) and proposed some methods for detecting them based on data features (signal gradient, variance, mean, etc.). In ref. [2], they used some of these solutions as the basis for defining a fault injection framework, producing benchmarking data. The survey in ref. [14] also identifies types of problematic datasets and sources of anomalies (the environment, the system, the communication and attacks). It also explains that anomalies have been addressed from several domains: statistical methods, time-series analysis, signal processing, spectral techniques, information theory and machine learning. The survey in ref. [6] identifies missing values as another failure and describes multiple approaches in the same fields mentioned, including also a few solutions for error correction.

There are solutions that have been designed to automatically clean data from sensor networks. In the case of ref. [1], the authors proposed a system in which cleaning follows five stages (point, smooth, merge, arbitrate and virtualize) using simple queries. In the case of outliers and faulty readings, they propose to eliminate measurements beyond a certain threshold and to use the mean and the standard deviation of nearby sensors.

Other solutions [15] propose a cleaning method of calculating an influence mean, which gives weights to sensor measurements (or removes them) depending on their reliability, based on the similarity of measurements. Such work mentions issues with Spearman correlation in other solutions and does not take into account that similarity might not need to be so high in terms of current value.

In the field of real-time streams, ref. [16] proposes an improved method based on a Kalman filter applied to time series, such that it corrects the received values with the predicted ones from the filter when a certain threshold is reached. It is focused on additive outliers and assumes that the sensor produces continuous values, and no big jumps are expected in the data. Moreover, variance is at the core of the model, as a key parameter to determine the acceptable boundaries. The work in ref. [14] also mentions other approaches in the area of time series analysis, such as auto regressive integrated moving average (ARIMA), but obtaining good models is very complex and problem specific; moreover, when detecting anomalies, they need to assume that the estimation is more accurate than the real value and, therefore, values far from the estimation are considered outliers.

Finally, there is another group of solutions that attempt to solve the problem by using machine learning techniques. The work in ref. [17] classifies solutions for anomaly detection focused on machine learning. Parametric and non-parametric solutions are mentioned. While the first group was mainly focused on detecting attacks and loss of data [18], the second one was focused on the detection of abnormal values, including solutions based on K-nearest neighbors (kNN) [19], support vector machines (SVMs) [20], artificial neural networks (ANNs) and genetic algorithms (GA). According to the study in ref. [21], it compared logistic regression (LR), SVM, decision tree (DT), random forest (RF), and ANN, finding as a result that the solution based on RF performed better in general. The applicability is huge, especially in the context of the Industrial Internet of Things (IIoT), where we may find solutions using some statistics for dispersion together with an unsupervised ML algorithm for anomaly detection [22] (for manufacturing), as well as solutions applying yet another segmentation algorithm (YASA) with a one-class SVM [23] (for the oil industry). This kind of solution also has been applied in the field of autonomous vehicles [24], detecting anomalies in sensors (in real-time) using a long short-term memory (LSTM) autoencoder that extracts data stream features and feeds a convolutional neural network (CNN) for classifying the anomalies.

The problem with ML-based solutions is that they tend to be too problem-specific, not being able to generalize for other systems and types of sensors. Additionally, parametric classifiers such as Gaussian Naive Bayes, linear discriminant analysis and quadratic discriminant analysis assume a normal distribution of the data (because of the way they use statistics such as the mean and the standard deviation). Therefore, it is important to understand if the data obtained from the sensors can fulfill such an assumption. Otherwise, it may be necessary to apply some transformation to the data (e.g., Box-Cox [25] and Yeo Johnson [26] transformations).

## 3. Research Methodology and Data Used

### 3.1. Research Methodology

The main research methodology followed during our work was the design science research methodology (DSRM), as presented by Hevner et al. [27] and Peffers et al. [28]. Our main objective was to build an artifact that would support data understanding, as part of a more complex system for determining the trustworthiness of sensors in IoT environments.

The steps that we followed when carrying out the research were those proposed by Peffers et al. [28], after analyzing several methodologies and guidelines to implement DSRM:

- *Activity 1. Problem identification and motivation*—As stated in the introduction, we believe that it is necessary to formalize data understanding processes and to provide a tool for supporting such formalization, based on statistical tests (as they can be fast and flexible), and applicable to multiple domains. We identified several research questions related to the approaches to use and the aspects to address (in Section 1). With such tools, it will be possible to understand how data are expected to behave, discarding solutions that might be affected by the nature of the data (e.g., as mentioned in Section 2, some ML-based algorithms could show problems when using datasets that are not following a normal distribution).
- *Activity 2. Define the objectives for a solution*—A set of objectives were defined, including the concrete aspects that must be addressed (variability, distribution of the data, presence of outliers and their effect), the application of multiple statistical solutions (we explore as many as possible), the applicability to different domains (using datasets from multiple domains was a must) and the efficiency of execution (therefore, the parallel implementation, as a way to experiment with execution in multiple cores, as available in edge environments and Cloud solutions). The solution must provide enough information for the users to make decisions on how to process the data.
- *Activity 3. Design and development*—This activity was the most complex one, applying a quantitative research method approach, in which certain experiments were carried out to analyze different statistical solutions, with the purpose of observing which solutions were working, what results they were providing and how they could be used. As a result, we identified the key aspects to include and designed the three processes to be implemented. Then, these processes were implemented with R scripts, applying parallelization to the code.
- *Activity 4. Demonstration*—As a way to demonstrate the validity of the solution, we carried out a complete analysis of a new dataset (with a new sensor type not studied before) using the implemented R scripts. We observed the information generated by the scripts by configuring the usage of two air quality sensors. The demonstration included the execution with different numbers of cores, showing how the implementation could be scaled up (10 executions were performed per script and core configuration).
- *Activity 5. Evaluation*—We observed that it was possible to quickly obtain valuable information about the characteristics of the data by using the scripts. We checked whether the information provided was accurate, including the identification of outliers (by comparing the generated results with the visual observation of the dataset, as well as other graphs such as histograms). Metrics such as F-score were available in some cases (e.g., in outliers detection). We also generated corresponding graphs for speedup and execution time, in order to observe the efficiency of parallel execution.
- *Activity 6. Communication*—Once we had results to communicate, we proceeded with the preparation of an article to explain the results from our research.

It is important to highlight that the method followed in Activity 3 included research based on primary and secondary quantitative research methods. In some cases, we were the ones generating some of the datasets (as in the case of data from smartphones and the sensors attached to the Arduino board) and, therefore, we had the control to influence the data obtained (such as generating certain types of outliers on purpose). In other cases (such as the Ports of Spain and air quality datasets) the data were provided by third parties and, therefore, we could only rely on the information they provided together with the data (such as annotations about data quality).

We carried out experimental research by writing R scripts (usually only focused on a concrete set of aspects, such as homogeneity) that analyzed different statistical solutions in different time windows (from basic statistical properties, such as average, range, variance and median, to statistical tests for outliers, homogeneity or trend detection) with the datasets we had access to, belonging to different domains (environmental monitoring, home automation, smartphones and smart cities). Such experiments randomly selected samples of two different longitudes and were used to answer the research questions about the utility of the methods, the aspects of the sensors that may influence their behavior, the data distributions we may find, the utility of correlation, the basic detection of outliers and their influence on other results. With the information generated, by induction, we identified the statistics to apply, how to use them and how to make them complementary. The R scripts we implemented were evaluated later (as explained) to determine if they were able to provide the expected results.

Taking these aspects into account, we can confirm that we fulfilled the six guidelines proposed by Hevner et al. [27]:

- *Design as an Artifact:* We produced a set of processes for data understanding and decision making, together with their (parallel) implementation;
- *Problem Relevance:* Our work addressed an important business problem of understanding the data before carrying out complex data analytics. The implemented scripts could support researchers and practitioners when selecting the most appropriate data analytics and ML solutions;
- *Design Evaluation:* We defined a way to evaluate several aspects of our results, including not only the capability to provide relevant information and accuracy of results, but also the performance when executed in parallel;
- *Research Contributions:* We identified several aspects that affect the characteristics of the sensors’ datasets, such as potential issues with variance, the reality about the probability distribution of the data, statistical tests that may be problematic, etc. This knowledge was used to implement a set of useful scripts, and we also demonstrated the utility of parallelization as a way to increase performance when analyzing sensors data;
- *Research Rigor:* As explained, we followed formal research methods to identify key aspects and to design the R scripts, while the evaluation method was also formally defined;
- *Design as a Search Process:* We used all possible means to obtain a useful solution, adding as many sensor types (as we had access or generated new datasets) and statistical solutions as possible, to contrast the results and gain more knowledge;
- *Communication of Research:* This article is a good representation of communication to a technology-oriented audience.

### 3.2. Data Sources

We used data from different sources in order to cover several types of sensors, locations, frequencies and providers, for the purpose of identifying several particularities and even differences in datasets for measuring the same aspect (i.e., temperature) in different places. The objective was to address several domains, such as smart cities, agriculture, home automation and environmental monitoring (e.g., weather).

Ports of Spain provided several datasets from the area of Algeciras port, covering meteorological stations, buoys, and tide gauges. They included data from several sensors, measuring sea level or wave height (in some datasets, it is an average of the last 24–26 min), wave direction (in some datasets, it is an average of the last 24–26 min), water temperature at the surface, speed of the ocean current (average of last 10 min), direction of the ocean current (average of last 10 min), atmospheric pressure, air temperature, wind speed (average of last 10 min), wind direction (average of last 10 min) and water salinity. These datasets included several years of data (in some cases, since 1996), and the collected information followed different frequencies depending on the dataset (from hourly data to measurements every 5 min). They also contain faulty values, represented as −99.9. The Ports of Spain website (http://www.puertos.es/en-us accessed on 29 August 2021) shows the locations of the devices used (separated by many kilometers in some cases) and provides access to some data.

Another source was the project HiDALGO (https://hidalgo-project.eu/ accessed on 29 August 2021), which provided data from Bosch air quality devices installed in the city of Gyor. These devices provided information about relative humidity, temperature, air pressure, NO_2_ concentration, O_3_ concentration, PM10 concentration and PM2.5 concentration, with measurements almost every minute. They cover one week of data, which provided measurements every minute. The devices were separated by distances ranging from hundreds of meters to several kilometers.

Additionally, the work described in ref. [2] generated some of the benchmarking datasets that were used. Such datasets included measurements of light intensity and temperature in three different scenarios: indoor light and temperature (controlled by an Intel-based platform), temperature from the SmartSantander platform (https://www.smartsantander.eu/ accessed on 29 August 2021) and outdoor temperature from a SensorScope platform [29]. The data (raw and interpolated) were used to analyze sensor behavior, while the datasets with fault injection (bias, drift, malfunction and a mixture thereof) were used to analyze how certain models behave when processing certain anomalies. These datasets included measurements over periods from one month to a month and a half, at intervals of a few minutes.

Using an Arduino board and some sensors (a hygrometer for moisture and a sensor for temperature and humidity), we built a small data collection solution, connected to the soil of a flowerpot located in a house. It contained data for several days, with measurements every 2 min. It also contained a few outliers on purpose (such as for irrigating the plant or changing the location of the hygrometer), for realistic analysis.

Finally, the last source was the Physics Toolbox Suite application (https://play.google.com/store/apps/details?id=com.chrystianvieyra.physicstoolboxsuite&hl=es_419&gl=US accessed on 29 August 2021), executed in an Android mobile phone. Thanks to such applications, it was possible to collect in real-time data from the following sensors: G force and linear accelerometer, gyroscope, barometer, proximity, magnetometer, luminosity and sonometer. Data from different sensors were exported to csv, with annotations for those situations in which something altered the measurement, whether natural (i.e., message received by the phone or somebody walking close to the light sensor) and on purpose (i.e., by shaking the phone for a few seconds). These datasets cover between 30 min and 1 h of measurements, at high frequencies and with no problems related to missing values.

## 4. Research Results

### 4.1. Sensor Types

Sensors behave differently. Although some works assume that measurements are, in general (and if everything is fine), continuous and do not contain large gaps in the data generated, this is not always true for all type of sensors.

**Figure 1:**
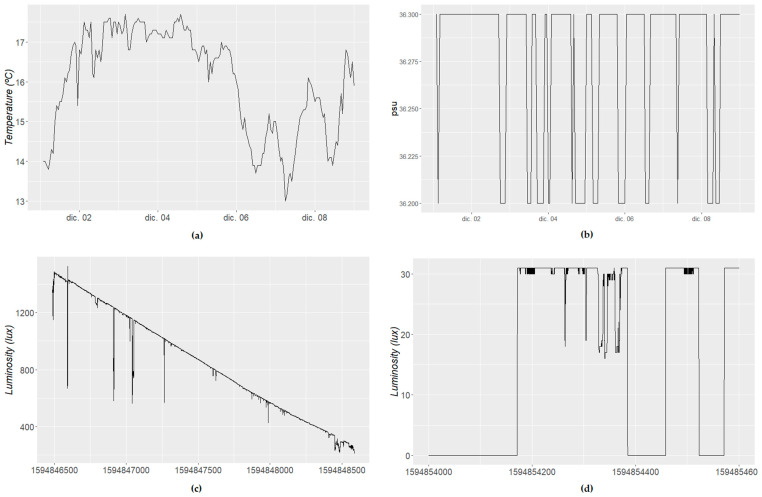
Behavior of different sensors: (**a**) Outdoor temperature sensor (1 week); (**b**) Salinity sensor (1 week); (**c**) Outdoor luminosity sensor during the afternoon (30 min); (**d**) Indoor luminosity sensor during nighttime (10 min).

As can be seen in Figure 1, water salinity sensors (we show 1 week of data in Figure 1b, which does not contain outliers) are quite consistent in their measurements, jumping suddenly to another level, where they spend some time, jumping again after some time. This might be problematic when performing certain filtering of outliers or when expecting certain variation. This also happens with moisture sensors from time to time, when the soil is irrigated.

On the other hand, temperature sensors (Figure 1a) do not show those jumps during normal operation, varying continuously and showing some seasonality (i.e., day–night, seasons of the year) as well as clear trends in short-term samples (a few hours of data).

In other cases, such as luminosity, we may observe large differences when operating indoors and outdoors. In the outdoor case (Figure 1c), the sensor behavior will be similar to that in the case of temperature, detecting daylight with some “outliers” (due to the shadows of people moving close to the sensor) and smooth variation. On the other hand, indoor sensors will show quite unstable behavior, especially during the evening hours, with unexpected peaks because of people turning a light on and turning it off later (Figure 1d). Their data will also show a few outliers when the lights were on, also because of shadows of people in the room.

This also has clear implications for the kind of distribution the data have. The distribution differs between sensors, although it also can be affected by other factors (such as frequency and time observed). In any case, this must be taken into account, as certain assumptions will make the models fail with certain kinds of sensors, removing outliers that should be kept (depending on the purpose of the data) and losing accuracy due to unexpected situations, even if there is some contextual information that may support the analysis.

### 4.2. Units of Measurement and Ranges

Depending on the type of real aspect to measure, we will use one sensor or another, which means that we are going to use different units of measurement (luminosity is measured in lux, acceleration in m/s^2^, humidity in mg/L, etc.). This means that the ranges of data that a humidity sensor generates have nothing to do with a sonometer.

Moreover, even measuring the same aspect, we use different units. For instance, temperature can be measured in °C, in °F or in Kelvin. In other cases, it happens that we use different scales, even with the same metric. This was the case, for instance, with the Ports of Spain and how atmospheric pressure was measured by different sensors. In some cases, it was measured in millibars (mbar or hPa), while in others it was measured in decapascals.

Statistics such as variance are highly affected by the ranges and scales and, in these cases, it means that variances will differ significantly between sensors, not only among different kinds of sensors, but also among sensors measuring the same event but with different scales.

For instance, data from different sensors measuring atmospheric pressure, from the Ports of Spain source, were used to calculate variance of the data. The calculation was performed for up to three stations (REMPOR Dique Abrigo, REDEX Golfo de Cádiz and REDCOSM Puerta Carnero) and for different time windows (full data, last year, last 6 months, last month, last 2 weeks, last week, last 3 days, last day, last 12 h, last 6 h, last 3 h, last 2 h, last hour, last 30 min). In all cases, as the number of used measurements increased, the variation increased as well (from 0.0039 to 13.35 in the case of Dique Abrigo).

When comparing variances in sensor data for the same periods (same dates), even using the same scale, sensors located not very far from each other (Dique Abrigo and Golfo de Cádiz) showed significant differences (i.e., 13.35 vs. 39.37 for last year, 0.33 vs. 0.92 for last day). However, the difference with Puerta Carnero was much larger, since that sensor was not using hPa, but decapascals. In that case, last year variance was 0.19 and last day variance was 0.0067.

Because of this effect, it is hard to define generic thresholds for those solutions that make use of variance in their models. Still, alternatives such as the coefficient of variation [30] could be helpful, because it is a unit-less metric useful for normalizing dispersion, although it also has its limitations (i.e., sensors such as those for temperature should be converted to Kelvin scale, as that is the only one with a meaningful zero value).

Another interesting approach is to analyze the interquartile range (IQR), which is calculated as Q_3_-Q_1_ (the difference between 75th and 25th percentiles) and provides an idea of the statistical dispersion of values. Because the outliers tended to be out of that range, we eliminated the anomalies they introduced (for instance, when calculating the mean), although the measurement units used will also affect IQR calculation (it is not a unit-less metric; hence, the same measurements in hPa or decapascals would result in different calculations of IQR).

### 4.3. Data Distribution

The statistical distribution of the data is relevant because it may affect the results of some statistical tests that are widely used when cleaning data and finding outliers. For instance, the standard normal homogeneity test (SNHT) [9], the Grubbs test [31] and the Pearson correlation coefficient [32] have been defined for datasets following a normal distribution. Therefore, not fulfilling that requirement may lead to faulty results when applying these solutions in the data pre-processing stage.

In order to analyze the distribution, two approaches were followed: a graphical one (with *Q*-*Q* plots and histograms) and a numeric one (Anderson–Darling test [33] for full datasets and Shapiro–Wilk tests [34] for 20 pieces of data randomly extracted from the dataset).

**Figure 2:**
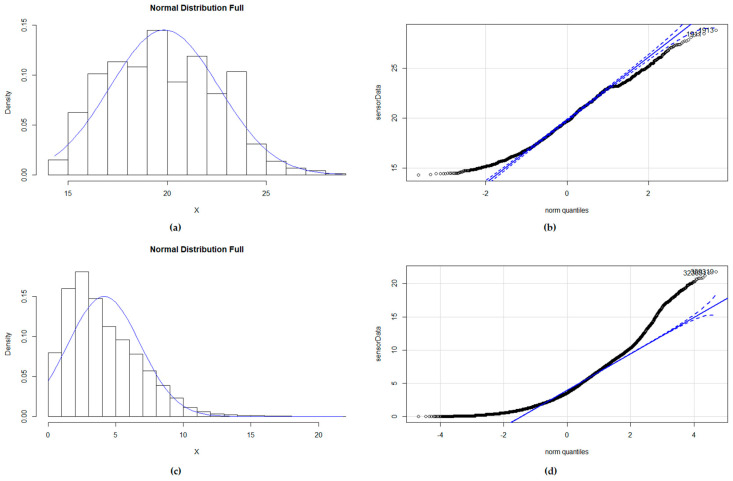
This figure shows the graphical analysis to determine if the datasets follow a normal distribution: (**a**) Histogram for a dataset with temperature measurements; (**b**) *Q*-*Q* plot of the same temperature dataset showing the deviation from a normal distribution (heavy tailed); (**c**) Histogram for the wind speed dataset; (**d**) *Q*-*Q* plot of the same wind speed dataset showing the deviation from a normal distribution (skewed right).

Analyzing the datasets, although some histograms showed that the distribution could be approximately normal (as in air pressure), the *Q*-*Q* plots and distribution fit experiments confirmed that this was not the case. In some cases (water temperature, soil moisture and water salinity), the distribution will be light or heavy tailed (values in the extremes are out of the expected part of the graph, and it seems the distribution is closer to a uniform one, as in Figure 2b). Other sensors show a skewed right distribution (as in Figure 2d), as values tend to concentrate at the right part of the mean (i.e., air temperature, sea level, water current speed, wind speed, precipitation, humidity). In many cases, they are close to a gamma, Burr or lognormal distribution (even Weibull in some cases).

When reducing the time window and, therefore, the number of measurements used, the data distribution was somewhat closer to a normal one. In the case of temperature, for instance, when taking time windows between 30 and 10 min, 60% of the selected data samples followed a normal distribution. This increased up to 75% when taking 10 min of temperature data.

Therefore, depending on the type of analysis to perform and the time window that is relevant for such analysis, it might be important to choose other ways to calculate mean and variance, and non-parametric solutions should be selected as the preferred ones.

**Figure 3:**
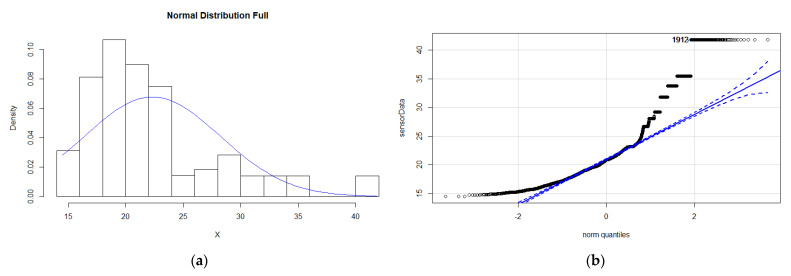
This figure shows the graphical analysis of data distribution for the Figure 2 temperature dataset with a bias fault injected: (**a**) Histogram of the temperature dataset; (**b**) *Q*-*Q* plot of the same temperature dataset, showing the deviation from a normal distribution (skewed right).

When errors appear in the data, the distribution may be affected, depending on the magnitude of the errors and outliers. When malfunction error is present, the normality may even improve (as observed with the datasets provided by ref. [2]), because it adds a few extreme values that support the Gaussian shape of the mean. On the contrary, bias errors are exemplified by the existence of many constant values that are overrepresented, making the dataset less normally distributed and skewing the distribution (see example for temperature using the same sensor as before in Figure 3). When drift error is present, the data become heavy tailed, increasing the weight of extreme values.

Consequently, even when using time windows in which the data may follow a normal distribution, if drift or bias errors are expected, non-parametric solutions should be preferred.

### 4.4. Outliers and Homogeneity

One of the most important aspects is the identification of outliers, because these are values that could alter the analysis of the data (i.e., with a relevant impact on means, variance, ranges, etc.). There are specific statistical tests for detecting outliers, although they have some limitations. The best known are Dixon’s *Q* test [35], Grubbs test [31] and the extreme studentized deviate (ESD) [36] test, a type of sequential application of the Grubbs test for more than one outlier. Although Dixon and Grubbs can detect one outlier, ESD was designed to detect multiple outliers (the Tietjen–Moore test [37] also can do it, but requires being provided with the number of outliers beforehand, so is not useful for our case).

Based on the experiments performed, Grubbs was able to detect outliers in a quite robust way (even with salinity measurements in most of the cases), although it showed problems when there was a drift error with change in the level of the time series (because that alters the mean and standard deviation calculation). It also exhibited issues when there was a strong trend in the data (usually in temperature), masking outliers, and may detect as outliers the lack of normality in the data. This is related to the usage of mean and standard deviation, which are affected when the number of outliers is high [38]. Dixon has the same issues and has two more of importance: it is limited to 30 points (because of the way the critical value for the *Q* statistic is calculated) and its *Q* statistic becomes 0 when the smallest points in the time series have the same value, because the numerator of the *Q* Equation (1) equals 0. Therefore, it is better to discard Dixon and focus on Grubbs and ESD tests for detecting outliers, transforming the dataset, if necessary, to eliminate trends and seasonality. For trend-related transformation, a differences function (such as ‘diff’ in R) is useful, although others such as polynomial regression can provide good results, especially when we have different trends in the same dataset. Removing seasonality requires more complex transformations.

(1) $$Q=\frac{x_{2}-x_{1}}{x_{n}-x_{1}}\;,$$

In many cases, homogeneity tests are used in order to determine if there are values that “break” the coherence of the dataset, detecting where this “jump” in the data happens. The most common ones are the standard normal homogeneity test (SNHT) [9] and Pettitt [10], although others such as the Buishand (range and U) [11,39] and Lanzante [40] are used as well. From this list, only the SNHT and Buishand claim to assume a normal distribution of the data; still, SNHT seems to yield good results with non-normal datasets, according to the experiments.

After using these tests with several types of sensors a, first conclusion is that, while Pettitt and Lanzante seem to be very similar, SNHT and Buishand also look quite similar, agreeing on the “change points” almost always. Although the graphs generated with the test statistic were extremely similar for Pettitt and Lanzante, there were some differences between the SNHT and Buishand tests.

As a limitation, the experiments showed that in datasets with strong trends, the tests may indicate that there is a change in the data level at the center of the time series. Therefore, again, it is important to detect trends (with Mann-Kendall [41]) and remove them.

**Figure 4:**
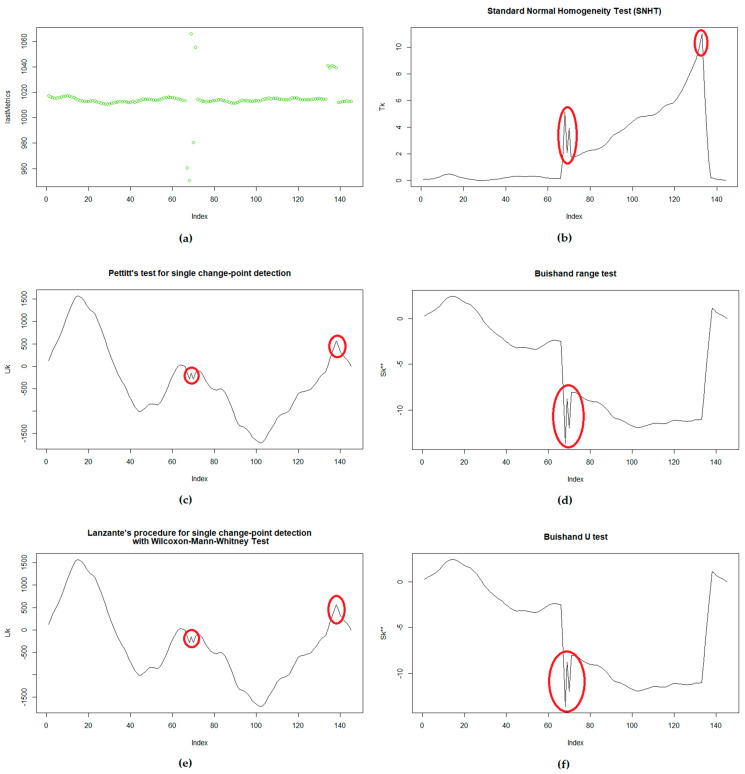
This shows an example of atmospheric pressure outlier detection with homogeneity tests: (**a**) Full dataset with atmospheric pressure metrics, with outliers; (**b**) Graph of the statistic calculated with SNHT; (**c**) Graph of the statistic calculated with Pettitt; (**d**) Graph of the statistic calculated with Buishand range test; (**e**) Graph of the statistic calculated with Lanzante’s test; (**f**) Graph of the statistic calculated with Buishand U test.

As an example, the graphs in Figure 4 show the usage of the tests with atmospheric pressure (6 days of data), in which a few outliers were introduced in order to illustrate some observations (in line with the way that is done in ref. [2]). First of all, SNHT, Pettitt and Lanzante detected both outliers very well, but whereas SNHT reaches its maximum value in the second graph (K = 133), Pettitt and Lanzante reach their maximum much earlier (K = 102), although in that position there is no change yet. Therefore, the three report that the dataset is not homogeneous (SNHT reports T = 11.043 and *p*-value = 0.02167, Pettitt reports U* = 1712 and *p*-value = 0.006501, and Lanzante reports W = 1337 and *p*-value = 0.00021). This is due to limitations in the results of the tests, but it is possible to see the peaks in the graphs clearly, whenever the outliers appear. We observed the same peaks with the Arduino dataset (in the temperature and humidity measurements, but not in those for moisture), in a case in which the outliers were generated by “vandalizing” the sensors for a few minutes (blowing heat into the device and moving the hygrometer to another location).

In the case of the Buishand range and U tests, both failed to report the anomaly (Buishand range reported R/sqrt(n) = 1.336 and *p*-value = 0.2403, while Buishand U reported U = 0.38263 and *p*-value = 0.07867), with a probable change point at k = 68. Still, looking at the graph of the calculated statistics, it is possible to see some “small peaks” in the positions in which the outliers were present.

The same experiment included Grubbs, ESD and Dixon tests in order to compare the solutions. Grubbs detected the outlier with value 950.5 (the lowest value) with G = 6.0336 and *p*-value = 7.073 × 10^−9^. The Dixon test was applied only to the middle of the dataset (due to its limitation) and detected the same outlier with *Q* = 0.45954 and *p*-value = 0.0188. The ESD test was executed with an upper limit of nine outliers and reported the nine correctly.

It is interesting to mention that, when testing different outlier injections and there were only three outliers in the drift error (the one detected at K = 133), the SNHT was not able to detect the anomaly (with a *p*-value of 0.387), while Pettitt and Lanzante still detected that something was wrong (although in K = 15). The same happened in other experiments (such as those performed with the Arduino dataset), in which the statistical tests pointed to different locations (the K result) as the position of the outlier.

This means that, instead of relying only on the final result of the tests, it would be better to go through the list of statistics calculated, looking for certain figures in the statistic value that may indicate more accurately where one or multiple outliers are present. Moreover, tests such as Grubbs, ESD and Dixon are better for detecting a small number of outliers (malfunction errors), while homogeneity tests are useful when the number of outliers is larger and they are concentrated (drift errors). Therefore, the tests can complement each other.

Additionally it, is important to highlight that, in almost all the cases, the homogeneity tests reported that some change was present, being too sensitive and causing many false positives. This might be because of the thresholds defined for the tests, which could need some customization. In that sense, it was possible to observe that the ranges of values of the statistics vary significantly depending on the type of sensor and the time window. While in some cases Pettitt yielded values between 1500 and −1500 (6 days of data on atmospheric pressure), in other cases it was between 0 and 200 (one-and-a-half days of atmospheric pressure data) or between 100 and −3000 (6 days of water salinity measurements). The same applied to all the statistics. Therefore, some normalization solution might be useful for generalizing the results.

Finally, it is important to highlight that the presence of outliers does not mean that there is a problem with the data. In the case of water salinity and light intensity (indoors), because of the characteristics of the data, some changes in the time series were considered a “jump” and reported as an outlier. The same issue occurred with moisture sensors, as irrigation was detected as an outlier. Although this is fine from a statistical point of view, the particularity should be considered, without removing or modifying such values.

### 4.5. Equivalent Sensors and Their Locations

As mentioned before, several solutions rely on the comparison of measurements between sensors of the same type in order to identify which ones are providing normal data and to use such consensus of metrics to clean and adapt the data to be stored. Works such as ref. [42] identified some general aspects on the use of correlation, but it is also interesting to investigate how it works for different sensors and datasets. In principle, it should be useful, as we could expect sensors in the same area to produce similar measurements, and that these evolve similarly over time, but caution should be exercised because when there is no consensus, it is very difficult to know which sensor (or group of sensors) is wrong.

Taking a look at data from Ports of Spain, the Dique Exento Norte and Dique Exento Sur weather stations are 1.12 km away and are of the same type. The experimentation consisted of taking 1 week of data and 1 day of data for two rounds of experiments, selecting the starting time randomly (but always using the same dates for both rounds).

As mentioned before, dataset distribution is important and, in this case, taking 1 week of data randomly showed that the data were not following a normal distribution (Shapiro–Wilk tests gave a *p*-value < 2.2 × 10^−16^ for Dique Exento Norte and *p*-value = 5.371 × 10^−15^ for Dique Exento Sur, and *Q*-*Q* plots showed a clear light tail). For the 1-day data, in some cases the tests determined that the data were following a normal distribution.

**Figure 5:**
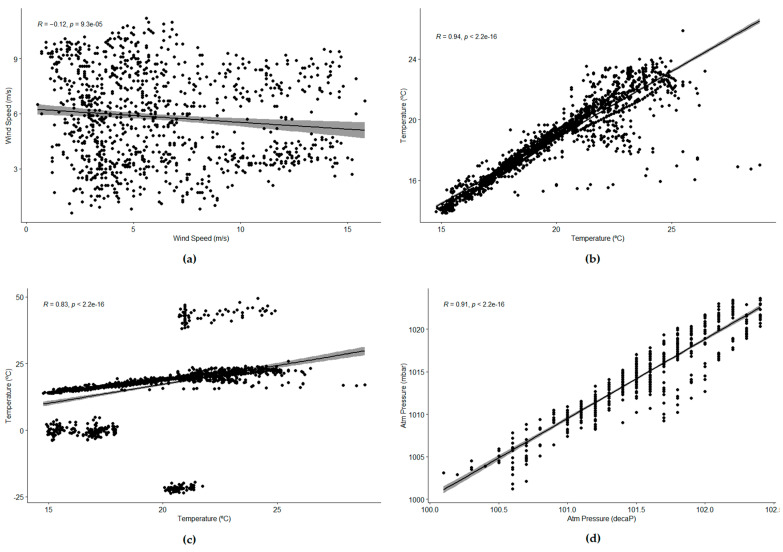
This figure shows the correlation diagram for different types of sensors and datasets: (**a**) Spearman correlation for wind speed (1 week); (**b**) Spearman correlation for temperature without errors (1 week); (**c**) Spearman correlation for temperature with drift errors (1 week); (**d**) Spearman correlation for atmospheric pressure (1 month), with different measurement units.

In order to determine similarity, three correlation tests were used: Pearson [32] (even if the data was not following a normal distribution), Kendall [43] and Spearman [44]. In all cases, the results were that there were no linear correlations between the datasets (all the indexes were close to 0), as also can be seen in one of the diagrams generated (see Figure 5a). In this case, the Pearson correlation index was −0.1081096, Kendall’s tau gave a value of −0.08060731, and Spearman rho was −0.1227435.

In the case of temperature, the experiments showed that, although the normality of the data was not true at all, the correlation tests could be used to determine that the data had a strong linear correlation (see Figure 5b,c). A few sensors from the Smart Santander infrastructure were selected, guaranteeing that they were located in the same area and were separated by around 30 m from each other. With 1 week of data, the Pearson, Kendall and Spearman tests provided values close to 1, showing a positive linear correlation.

Because guides on the correlation tests indicated that Pearson might be sensitive to outliers in the data, we took advantage of the benchmarking datasets provided by ref. [2], performing the same analysis of datasets in which one of the sensors had some errors injected. The same experiment was performed with each type of error, obtaining the results shown in Table 1; hence, it is possible to compare the indexes in a situation without error and with different errors.

Comparing the concrete results of one of the experiments, it was possible to see that all the correlation tests were quite robust when there was a malfunction error (a few random values with some small peaks). In the case of bias errors (constant value breaking the continuity of the measurements for a short period), all reduced the linear correlation strength, although the results may still have been interpreted as having good correlations, especially when looking at the Kendall tau and Spearman rho (Pearsons’ index behaved worse). However, the largest difference was when there was a drift error (outliers with peaks and several dispersed values during a short period of time); in such cases, Kendall tau and Spearman rho showed that a linear correlation was there, but the Pearson index decreased significantly and was closer to 0, indicating that there was no correlation. Therefore, if we expect to find outliers and errors in the data, Pearson may not be the best choice.

When analyzing data for a single day, again, linear correlation is important under normal conditions but, depending on the number of errors injected and their duration, the results may be worse for any kind of error. With a large number of measurements, the errors were diluted somewhat more by the rest of the data but, as the number of measurements decreases, any error has a larger impact.

Finally, another experiment with atmospheric pressure showed that the scale used was not so relevant and that some events were correlated even over large distances (see Figure 5d). The experiment used data from Ports of Spain, from the buoy in Puerta Carnero and the buoy in Golfo de Cádiz (more than 100 km away). This time, we tried with 1 month of data, selecting starting dates randomly again, for 10 groups of data. Again, the data seemed not to be following a normal distribution (although for the case of Golfo de Cádiz buoy, it was close to normal).

The Pearson, Kendall and Spearman tests determined that there was a strong linear correlation, although it was interesting to see that, this time, the consensus was not so high. In the case shown in the figure, the Pearson index was 0.9276692 and Spearman rho was 0.9108291, while Kendall tau was 0.7908868. Comparing the graphs with those obtained for temperature, in this case, Pearson and Spearman seemed to be a bit overestimated.

**Figure 6:**
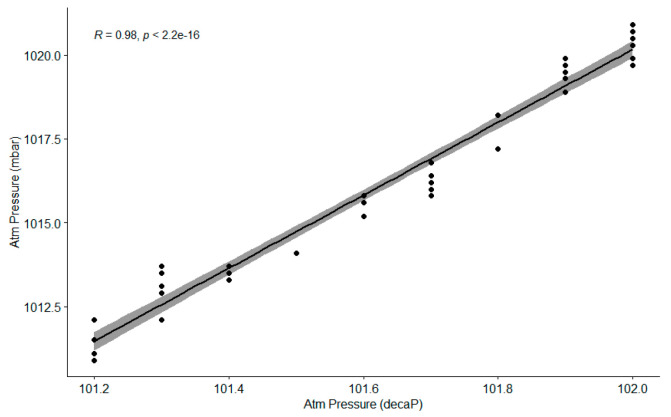
Spearman correlation diagram for atmospheric pressure (2 days).

When we performed the same experiment with 2 days of data (measurements are taken hourly, so the time series for 1 day is rather short), the results were very similar. Again, the data did not follow a normal distribution (although in some cases they almost followed such a distribution, according to the tests and the *Q*-*Q* plots), and there was a strong linear correlation between both sensors. In the example shown in Figure 6, the Pearson index was 0.9864971 and Spearman rho was 0.9791508, while Kendall tau was 0.9220732. The consensus among the three tests was even stronger than before, showing again that the Pearson index is not affected that much by the use of non-normally distributed datasets.

Therefore, it seems that for the analysis of similarity and consensus between sensors, the time windows and scales used were not so relevant. It was more relevant to take into account the type of sensor we used. Although the location is also important, unless it alters the sensor in particular, some correlation should still be there.

### 4.6. Proposal for a Decision-Making Procedure

Considering the analysis performed, and as a summary, we defined a few decision processes in order to select the most appropriate way to proceed with the collected sensor data during the data understanding phase; these processes were designed to be generic enough to be applicable to many kinds of sensors.

The basis for defining such processes was related to existing methodologies defined for data mining and related activities. This work took into account the cross-industry standard process for data mining (CRISP-DM) methodology [45] and the proposed extension described in ref. [46], which is more focused on engineering domains (where IoT is very relevant).

Data understanding and data preparation are the main phases in which this work was focused, so scientists and practitioners will know how to deal with the data. The data understanding phase was where the first analysis of the data was carried out, in order to understand the knowledge, the content, the initial hypotheses and the potential quality problems that may arise. Data preparation instead deals with tasks such as data cleaning. Thanks to the analysis performed in this work, it was possible to perform an initial analysis of the data with a deeper knowledge of IoT while also supporting the definition of a good approach for the implementation of data cleaning tasks (i.e., outlier detection).

In general, it is important to always start by selecting the appropriate time window, depending on the problem we are dealing with. If we want to analyze what happened in the data we stored (to be used in machine learning-based solutions), a good option would be to analyze the data as a whole and then to select samples of different lengths, so it is possible to analyze the behavior of the sensor in different contexts. In the case of outliers and data streams, it is interesting to use sliding windows, so that there is constant analysis of the data. The longitude of the sliding window is not usually long because data used in real-time is usually related to quick decision-making (as in SCADA systems).

**Figure 7:**
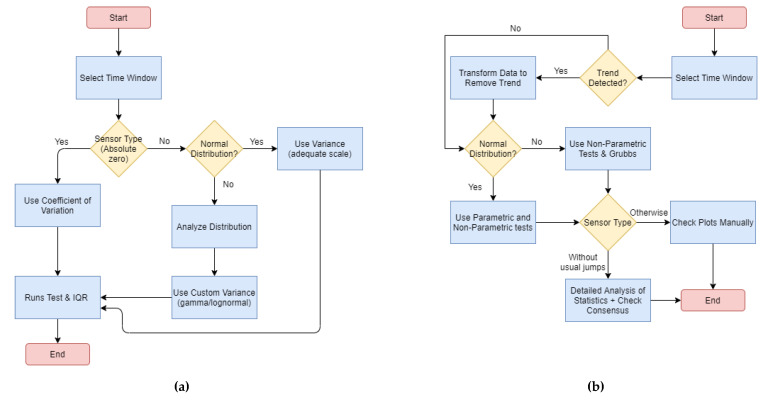
Processes for analyzing data from sensors: (**a**) Process to analyze variation of data; (**b**) Process for analyzing the presence of outliers in the data.

First, the process of variation analysis (Figure 7a) is focused on understanding how the data is spread and could help to understand if data is being created randomly or if there is a bias error. It is important to see if the coefficient of variation is applicable (only to those units with absolute zero, such as lux or Kelvin) and to know the data distribution so that we will apply the right variance formulas. We can use *Q*-*Q* plots, Shapiro–Wilk and Anderson–Darling tests to check the normality, but it is necessary to fit the data to other distributions and to analyze the goodness of fit for them [47]. At this stage, we checked gamma, Weibull, normal, uniform and lognormal distributions, as they were the ones detected in previous experiments with other sensors. The runs test was applied to determine if the data seemed to be random and IQR also could provide an idea of the variance of the data.

The process for analyzing outliers (Figure 7b) is focused on the detection of unexpected peaks in the data, addressing drift and malfunction errors. First, we must determine if there is a strong trend in the data and eliminate it (through a transformation), so the chance of detecting outliers correctly increases. Periodicity of the data may also be problematic (although the solution is more focused on short datasets), so a polynomial regression might be necessary. Then, it is important that we know if the data follows a normal distribution, so parametric (SNHT, Grubbs, Buishand range and Buishand U) and/or non-parametric tests (Pettitt and Lanzante) will be applied, although SNHT and Grubbs (as well as ESD) also can give good results in most cases. Looking at the results of all the tests, it is possible to compare the results and decide whether to accept the existence of outliers or not. Additionally, depending on the type of sensor, it might be necessary to perform a manual check because some sensors may generate misleading results in the tests (e.g., salinity and indoor luminosity).

**Figure 8:**
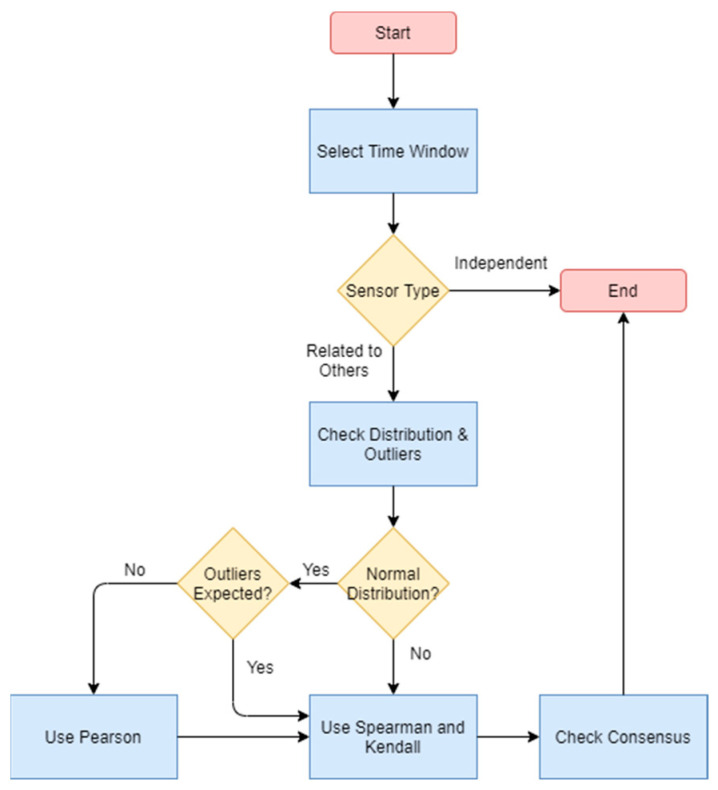
Process for analyzing correlation with other sensors.

Finally, the analysis of correlation (Figure 8) is interesting to understand if we can analyze several sensors together, performing data fusion or checking malfunctions based on consensus solutions. When applying sensor equivalence, it is important to understand that it is not applicable for some sensors and that, once we decide it can be applied, we should select the appropriate test depending on the data distribution and on the existence of outliers (since Pearson seems to lose much of its efficiency when they are present). Because the process is for identifying the relationship, in case of doubt, it is better to execute the whole process.

These procedures have been implemented as R scripts (one script per process) that read the data in CSV format and apply different statistical tests (using the libraries nortest, trend, outliers, EnvStats, fitdistrplus and randtests), storing the results in a file and saving graphs from the selected data and the statistics (when possible). Since nowadays almost any device has multiple cores, the code was implemented in such a way that it can be executed by parallelizing the calculations in different cores, through the %dopar% feature (from the *foreach* *and doParallel* libraries). Taking into account the increasing number of sensors and data available, as well as the computational capabilities of new systems and devices, it is important to understand whether it is possible to reduce execution times, thus facilitating the inclusion of more complex pre-processing features, especially for edge devices (allowing them to apply some data cleansing and filtering before sending data to complex analytical tasks).

The implementation selects several main indexes in a random way, as the point to start extracting data samples. Then, from those indexes, it generates a sliding window with the number of steps selected, having as a result a nested iteration that analyzes all the window steps as if they were “screenshots” of the sensor data (in the same way a complex event processing engine would do). The parallelization has been applied to the outer iteration, so each core analyzes all of the complete sliding window of a main index. This allows the CPUs to take advantage of their cache memory, while giving each core enough computational complexity to benefit from the parallelization (otherwise, the overhead for managing parallelization would be too high compared to the calculation time). Spreading each “screenshot” of each sliding window among the different cores might require that the CPUs access the main memory many times because the data is not already stored in their L1 or L2 caches, whereas handling the process in the same core takes advantage of the existence of all the data values already loaded into cache memory, except one.

### 4.7. Evaluation Applying the Processes

**Figure 9:**
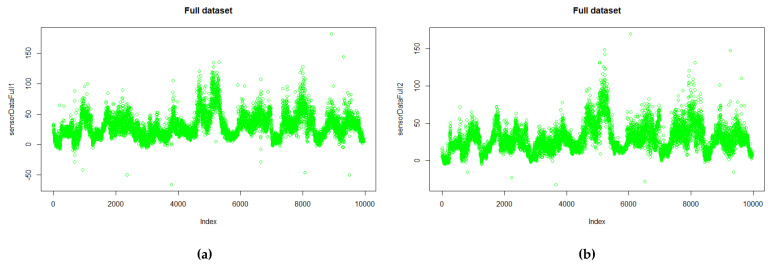
Datasets used for analysis with NO_2_ measurements: (**a**) Sensor 1 measurements; (**b**) Sensor 2 measurements.

To validate the proposed solution, we used the data from air quality sensors in order to check the variation of the data, if outlier detection worked normally, and if there were any correlation between sensors. The experiment consisted of the selection of two of the devices (which are located in the center of the city, at a distance of 750 m) and the analysis of the NO_2_ sensor (Figure 9 shows the measures), which is a type of metric not studied before. Long and short windows of data, as well as sliding windows, were used in order to analyze the data. While the size of the long windows covered 6% (10 h) of the total amount of data, the size of the short windows covered only 1% (75 min of data).

#### 4.7.1. Variation Analysis Process

For the study of variation, the two datasets were studied independently, running the script three times for each. This experiment generated, for each sensor, 12 long data windows (sliding the window 50 positions) and 20 short data windows (sliding the window 15 positions), with a total of 2700 results.

In the case of the first sensor, for long windows, none of the tests confirmed a normal distribution, although when fitting the data to a known distribution, the normal was the one chosen in most cases (794), followed by the lognormal (468) and gamma (334). There were many data chunks also reporting gamma and Weibull as the best distributions, but none of them selected the uniform one. When using short windows, a few tests confirmed the existence of a normal distribution. For the rest, the one most selected was gamma, followed by lognormal (246 and 216, respectively).

The analysis of the second sensor, with long windows, reported that the data were never following a normal distribution according to the statistical tests, and when fitting with other distributions, the gamma distribution was the one that yielded the best results, followed by Weibull (985 vs. 321 times). The lognormal and normal distributions were also the best fit in several cases, but the uniform distribution was never selected. In the case of short windows, some tests confirmed a normal distribution, and the distribution fit selected the normal as the best one, followed by the lognormal (298 vs. 213), Weibull (177) and gamma (172) distributions.

Because the concentration of NO_2_ is measured in µg/m^3^, we considered that it has an absolute zero. Therefore, the coefficient of variation (CV) was also calculated. For the first sensor, the CV ranged from 0.27 to 0.77 in long windows and from 0.20 to 1.07 in short windows (although around 2/3 of the values were below 0.50). The CV mean was 0.49 for long windows and 0.44 for the short ones, while the median was 0.48 and 0.38, respectively. For the second sensor, values ranged from 0.31 to 0.73 in long windows, although high values were unusual, and most experiments reported coefficients of variation between 0.50 and 0.35, with a mean of 0.45 and a median of 0.40. In the case of short windows, CV went from 0.19 to 1.32 (in a few cases with extreme variation), with a mean of 0.42 and a median of 0.35.

With respect to the IQR value, in the first sensor it ranged from 9.49 to 36 for long windows, and from 5.86 to 45.34 for short windows (with 2/3 of the cases below 20). Mean IQR was 18.96 for long windows and 17.09 in short ones, while the medians were 17.28 and 17.09, respectively. In the case of the second sensor, it ranged from 7.96 to 46.24, although such extreme values were very unusual, with most IQRs between 15 and 25, a mean of 19.87 and a median of 18.19. For short windows, IQR went from 5.09 to 42.99 (with just a few cases above 25.0), with a mean of 14.54 and a median of 12.94.

In general, we can say the variation was a bit high. The calculated CVs were in line with the CV for an outdoor luminosity sensor with some outliers (around 0.42). In the case of IQR, the calculated values were higher than in sensors for temperature, and much higher than those for salinity (where IQRs reported just 0.1 in 4 days of data). Moreover, thanks to this process, we learned that the data do not really follow a normal distribution and gamma is preferred in most cases for window lengths applicable to real-time analysis.

#### 4.7.2. Outlier Analysis Process

For outlier analysis, the process used 10 long data windows (sliding 30 positions) and 15 short windows (sliding 15 positions), and it was executed twice. For the first sensor, with long windows, Grubbs reported the existence of outliers in all the data chunks (including the sliding windows), except in one case, in which the homogeneity tests reported the potential existence of change in the level of the time series. Looking at the graphs, it is true that there were many outliers spread in the time series. With short windows, only 1/3 of the cases reported no outliers. The Grubbs test was, again, the one that reported most of the outliers (275 out of 327), going from very clear ones (with *p*-value 1.07 × 10^−12^) to some near the limit of the test (with *p*-value 0.0489). In cases marked by doubts, the use of sliding windows supported the detection of some outliers (because they became more evident). The process was quite accurate with outlier detection, failing only four times (compared to visual checks). Therefore, its accuracy was calculated as 0.9 and its F1-score was 0.77.

Analyzing the second sensor with long windows, just a few cases were free of outliers (17 out of 600). In most of the cases (570) the Grubbs test (with its *p*-value ranging from 0 to 0.0491) detected the outliers correctly because there were many of them. In only one of the cases (including with sliding windows), homogeneity tests detected the main problem (which looked like a drift error in the data). When using short windows, only 80 out of 450 cases reported no outliers in the data. Grubbs, again, performed as the main test detecting outliers (in 312 cases), with *p*-values ranging from 9.06 × 10^−12^ to 0.048 (for the detected outliers), and reported very few false positives (compared to test results with visual inspection). The homogeneity tests reported some false positives as well. In general, the sliding windows worked fine, with values varying as expected for the detection, and only failed in four cases. In this case, the accuracy was 0.9 and F1-score was 0.77 (although precision and recall differed from the first case).

The conclusion of the outliers analysis was that these datasets contain many of them, especially individual peaks that sometimes were quite evidently errors (e.g., negative values such as −66.3), while in other cases it was not so clear, and perhaps automatically removing them would not be a good approach. In any case, the datasets required a careful cleaning.

Because the datasets did not contain drift errors, Grubbs was the most useful approach, and the homogeneity tests provided a few false positives. Therefore, it would be interesting to check if the results would improve using weights or a fuzzy model with the test outcomes, in order to give more importance to some of the concrete ones, while perhaps the use of polynomial regression, instead of the R ‘diff’ function, could also provide better results when transforming the data. This last modification is important if the selected window size includes some periodicity in the dataset, as it could mask outliers. Another aspect to analyze would be an implementation of the recursive extreme studentized deviate test (R-ESD) [48], which claims to outperform the seasonal hybrid extreme studentized deviate (SH-ESD) [49] test from Twitter.

#### 4.7.3. Correlation Analysis Process

When analyzing correlations, the implemented process used 12 long data windows sliding the window 50 positions and 15 short data windows with sliding windows of 15 steps, and it was executed three times (for a total of 1800 experiments).

The correlation tests with long windows reported that 1072 experiments detected correlations, while 728 did not. Moreover, while the maximum value of the Spearman test was 0.8819, the maximum value of the Kendall test was only 0.6898. The minimum values were 0.046 and 0.0321, respectively. It is interesting to highlight that in 494 out of 1800 cases, the Spearman rho was higher than 0.75, whereas, in the case of Kendall, in 632 cases the tau statistic was higher than 0.50. When calculating with the full datasets, Spearman’s rho was 0.659, while Kendall’s tau was 0.4764. Pearson was never computed because no normal distributions were detected.

In the case of short windows, the results were even worse. Out of 675 cases, 594 reported that there was no correlation (so only 81 cases detected correlation). The minimum values of the statistics were −0.371 for Spearman rho and −0.2627 for Kendall tau. In these experiments, only 15 cases provided a Spearman rho higher than 0.75 and a Kendall tau higher than 0.5. Pearson was computed only a few times (because of the small number of cases following a normal distribution). Nonetheless, due to the large number of outliers detected before, it was ignored (although it did not show relevant differences with the Kendall’s and Spearman’s tests).

This analysis showed that the correlations were very weak (because values were not so close to 1), or even nonexistent in some parts of the data. In the short datasets (1 h of data), there was no correlation in most of the cases. Even if there were many outliers (similarly to the malfunction error), in the case of a good correlation, Spearman and Kendall values should not be so low (as we saw in Table 1). It makes sense because of the type of sensor, how pollution is accumulated in certain areas and how it moves (hence, there might be some delay in the correlation, being more effective to use longer windows), so we have to be very careful if we want to use solutions based on correlation when dealing with the data, as they might not be adequate.

#### 4.7.4. Performance Analysis

Because the R scripts were implemented with parallelization taken into account, it was important to also analyze the results related to performance and determine if such parallelization made sense in this kind of problem, following the guidelines described in ref. [50].

The scripts were executed on a laptop equipped with an Intel^®^ Core i5-8350U vPro processor (four physical cores at 1.70 GHz and 3.60 GHz in turbo mode, 6 MB smart cache and a bus speed of 4 GT/s) and 8 GB DDR4 2400 MHz RAM. The software used was a Windows 10 Enterprise (compilation 19041.1052) operating system with R version 3.6.2.

For the analysis, the three implemented scripts were executed 10 times independently, using as input the same datasets with air quality data that were utilized before. The measurements were taken with the “system.time” function (we used the elapsed time property), that covered the entire code, except the initial load of the CSV file with the full dataset (because in a real-time system, such information might be available through streams).

**Figure 10:**
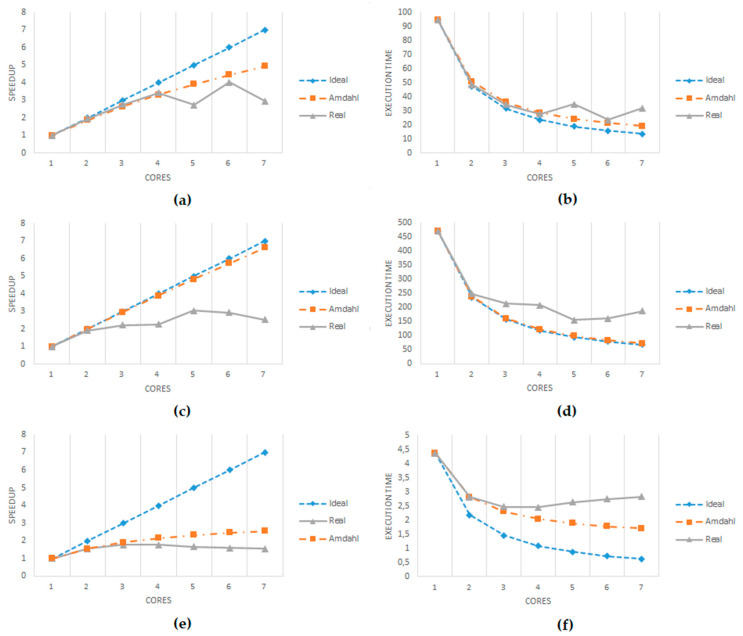
Graphs showing the performance of the parallel version of the scripts. (**a**) Speedup for the variation script. (**b**) Execution time for the variation script. (**c**) Speedup for the outlier script. (**d**) Execution time for the outlier script. (**e**) Speedup for the correlation script. (**f**) Execution time for the correlation script.

As we can see, the use of a high number of cores was not always synonymous with better performance (see Table 2 and Figure 10). Because there were only four physical cores (and eight virtual threads), the operating system and the application became saturated. The maximum configuration was with seven cores, because selecting eight could seriously affect all performance of the system. Still, when using six or seven cores, there were some issues, with the system failing, the R engine going down, and even the system reporting issues with memory allocation.

The graphs in Figure 10 illustrate the scalability obtained with the three scripts. While the graphs at the left show the speedup compared to the linear ideal situation and the serial overhead ideal (determined by Amdahl’s equation [51]), the graphs at right show the execution time, also comparing the real result with the linear ideal scalability and the serial overhead ideal (again, based on Amdahl’s equation, using as the base the serial execution time). The linear ideal assumes that all the code would be running perfectly in parallel in all the cores (1/number of cores). On the other hand, Amdahl’s equation differentiates between the code that cannot run in parallel (serial code) and the estimated parallel code, providing a more realistic ideal boundary. This analysis used the same dataset for all the runs, so we fulfilled the requirement to use Amdahl’s equation (2), where *pctPar* was the estimated percentage of parallel code and *p* the number of threads/cores used. The percentage of parallel code was estimated by measuring the serial and parallel parts of the code in 10 executions of the non-parallelized version of the scripts and calculating an average among those 10 executions.

(2) $$Speedup=\frac{1}{\left(1-pctPar\right)+\frac{pctPar}{p}}\;,$$

It is interesting to see that all the cases improved very well using two cores, and using three still yielded very good results in scalability. Then, only the variation script kept scaling well with four cores. With more than four cores, in most of the cases, the scalability declined (even worse than with fewer cores), as mentioned before. Testing with CPUs providing more cores would clarify if having more physical cores available still facilitates better scalability, or if there is some limit imposed by the code itself.

It is also worth mentioning the case of the correlation script, where around 71% of the code was estimated to be parallel (compared to the 99% in the outlier script and the 93% in the variation script). It was much more limited in the scalability it could reach because of that 29% of serial code. Therefore, it did not make sense to use more than three cores, unless the complexity of the calculations was increased (using four or more cores took more time to execute).

In any case, applying parallelization improves the execution time of the scripts. This is very relevant, especially for real-time environments, in which a faster response allows one to apply more complex calculations for data filtering and failure detection, adding more functionality at edge devices (so that data can be cleaned in early stages and some mitigation actions can be applied faster in cases of failure, such as in autonomous vehicles), although other offline systems (such as Cloud and HPC environments) can also reduce execution times drastically, making more efficient use of resources.

Finally, as Figure 10a,b shown, the variation script provided real measurements that were somewhat better than the boundary defined by Amdahl’s law. Even if the difference was very low (only 0.1), this may happen because the parallel execution of the *foreach* iterations does not print on-screen some of the information that the serial version does. Additionally, this is not unusual because memory and hard drive caching mechanisms sometimes may improve the measurements taken.

## 5. Discussion and Conclusions

This work analyzed different aspects of data generated by sensors that may impact the efficiency of applying certain solutions for data processing. As mentioned previously, some ML-based solutions (parametric classifiers) simplify the data processing, while adding certain assumptions (such as the distribution of the data). Further, as the presence of outliers may be misleading for the solutions, the proposed processes are useful to understand the data from sensors.

Both researchers who are developing models and dealing with data (e.g., complex solutions for repairing errors in the data, such as anomalies and missing values) and practitioners who want to process data to solve a concrete problem can benefit from this solution because it may save time and allow them to omit the application of certain methods in the early stages of the data preparation phase or determine which transformations to apply to their data as quickly as possible.

### 5.1. Particularities of Sensor Data

The type of natural element or property measured, the context of sensors, the measurement units and the time window used all affect the data generated and the basic statistical features that are widely used (e.g., mean, variance). This is not new at all, but we wanted to see the particularities of different sensors, as it would be easier to understand the results obtained from some analyses (such as the outliers, as some “false positives” may not be wrong at all). Looking at the datasets, it was evident that providing generic solutions would be quite difficult, as a solution that may work for temperature would fail with water salinity.

Using unit-less solutions, such as the coefficient of variation, is useful but they are not applicable in all cases (because of the lack of absolute zero or because the application of the typical statistics requires complex processing). IQR is also interesting because it may avoid the effects introduced by outliers, although it is not unit-less. There might be an interesting area of research applying these types of solutions instead of variance or standard deviation, to certain parametric models (both statistical methods and ML methods).

A key aspect is the analysis of the probability distribution of the data. Although many works using large datasets with average values (using yearly or monthly means) reported (or assumed) that the data followed a normal distribution (e.g., in the case of temperature [7]), the observations show that raw data, in many cases, does not follow normal distributions and, therefore, the assumptions of some models and solutions would not be right.

The selected time windows also have an impact on the distribution of the data. One of our observations was that, even using the same time window, different samples taken from the same dataset also followed different distributions. Therefore, the methods selected to apply may appear to be suitable for part of the data, but not for all of the samples we may select. We have highlighted how several sensors follow certain distributions (lognormal, Weibull and gamma were the ones selected most of the time, aside from the normal distribution). Because of so much variation, the use of non-parametric solutions is recommended unless the selected distribution is the one fitting in a high percentage of cases (only in one experiment did we observe a distribution as the one selected in more than 50% of the samples, and that was the gamma distribution). Still, in the cases in which the distribution appears close to normal (although statistical tests provide a negative answer, *Q*-*Q* plots and histograms provide good support in this case), the tools and methods to apply might still be valid (although perhaps they will not provide the best accuracy). In cases where applicable, another option that could be explored would be to propose solutions that adapt themselves as the distribution changes.

In some cases, it is hard to find complete information about this kind of characteristic of the data when looking at the statistical and ML-based solutions listed in Section 2 (the surveys in [6] and [14] mention potential issues with semi-supervised learning techniques, but do not mention the distributions we may find), so it is not so clear that they are given enough importance (such as in refs. [21,22,52], to name a few). In some cases, authors apply some previous transformation to the data that may solve the problem, but we do not know about the original conditions of the data, as in ref. [24]. Additionally, removing trends and seasonality is also shown as a solution [48], but we could still obtain datasets with non-normal distributions (such as a uniform distribution). Therefore, we consider this work useful because it is important for data scientists using sensors to address this aspect as soon as possible in their work, identifying appropriate strategies early in the process (the work in ref. [53] notes some issues when using non-normal datasets with ANNs and data distribution that should be taken into account when normalizing the input values for the models).

Another aspect addressed is the detection of outliers and their role in other characteristics of the data. As mentioned in Section 4.3, outliers may affect the data distribution by adding extreme values that can modify the original distribution. Therefore, we believe that a good way to proceed when preparing data would be to analyze the data distribution first (to understand if the application of certain methods for managing outliers might not be appropriate), then detect outliers (cleaning those that are real errors) and analyze the data distribution again as the last step.

In the case of outlier detection, as we sought to perform preliminary data processing, we proposed using certain statistical tests that can give a good idea regarding their presence (requiring transformation of the data in cases where trends and seasonality are present). Although there are other solutions that might be more appropriate in each case (e.g., ML-based solutions [22,23,24] can be more accurate in the concrete context they were designed for, but they can be affected by imbalanced datasets and require re-training and more analysis for new contexts), the statistical tests seemed to work fine, in general, in all the datasets we used, and more complex models can be applied later.

We observed that solutions such as Dixon’s *Q* were not adequate because of the potential issues with equal values. On the other hand, Grubbs and ESD worked fine when detecting small peaks. When multiple wrong values were together (drift failures), homogeneity tests demonstrated better performance (whereas Grubbs and ESD were missing outliers). Therefore, we applied a combination of both to detect most of these failures, instead of using only one, as proposed in previous works.

In fact, in the case of homogeneity tests, it was interesting to observe the presence of peaks in the position of the outliers when we generated a plot with the calculated statistics (SNHT T, Buishand R and U, Pettitt U and Lanzante U). Even in cases in which they provided a different position for the outlier, the peak was in the same location in the plot, although other values, in the end, set the reference value for the statistic. Although it did not happen in all cases, in those in which the peaks appeared, it would be possible to improve the accuracy of the result, detecting such patterns and complementing the information provided by the tests. This would be a new approach to outlier detection.

The last aspect addressed is related to correlation, a method used in several solutions for anomaly detection that exploits the comparison with other equivalent sensors [15]. As we have seen, correlation needs to be analyzed case by case because it is not applicable to all types of sensors (so cannot be generalized). While in some cases it will work even for sensors separated by kilometers (such as those monitoring atmospheric pressure), others do not show correlation even when they are close (such as with air quality and wind speed).

Due to the robustness shown with outliers, Spearman is the best solution to apply (despite the potential issues mentioned by ref. [15]), but it is always interesting to also have Kendall and Pearson to compare results. Although they can be useful for comparing sensors, we must be aware that using Pearson when drift errors are present may cause the solution to fail. This may affect some solutions focused on principal component analysis (PCA) techniques.

Correlation also can be used when comparing sensors of different types, when we think they are related, in such a way that we can better understand the context of each sensor. This needs to be analyzed case by case as well because not all types of sensors are correlated in every environment. Although we did not include this aspect in the processes we defined, it would be possible to include some correlation analysis for datasets with multiple sensors (all vs. all), in order to indicate which relationships should be analyzed more carefully.

### 5.2. Proposed Processes and Their Implementation

This article proposed following three processes to gain insights into sensor data before applying cleansing mechanisms or using the data. These processes addressed data variation (including data distribution), outliers in the data and correlation between similar sensors. Such processes can be seen as a way to specify and implement certain steps that are part of the CRISP-DM methodology. They fit very well with the data understanding phase, and our implementation can automate part of it, reducing the time spent by data scientists working with sensor data, thus enabling them to focus on their models.

The analysis of a new type of sensor (for air quality) showed that these processes can work for new cases, and they provide very relevant information, although some improvements could be explored, for example, by studying more distributions and applying other transformations to the data (such as polynomial regression or Fourier transforms for the seasonality of data). Therefore, although we cannot generalize to all kinds of domains, we can claim that our proposed approach is valid in sectors such as home automation, environmental monitoring, agriculture and smart cities. We understand that some domains, such as industrial environments, may have specific characteristics; therefore, we will continue to analyze more types of sensors from more domains.

The best way to implement the processes was through R script because of the strong support for the statistical methods available in this language (as multiple libraries are available). It would be interesting to compare implementations with other languages such as Python (also with good support for data processing), in order to analyze their performance and potential integration with other existing solutions.

Additionally, a parallel implementation of the processes was proposed to improve the performance of the solution. As more and more devices become available, more data streams will be available, and the computational resource requirements will be higher. Exploiting parallel processing is a step towards higher efficiency in the use of resources, already available in edge devices, as well as Cloud and high performance computing (HPC) environments. Although other works focused only on the solution itself, we considered this to be an important point to address for enabling new capabilities.

As a way to facilitate data processing in Cloud environments, for example, it would be possible to exploit multiple cores available in edge devices in order to perform a real-time preliminary analysis of data, annotating the data that arrives to the processing environment and facilitating complex data analysis. It could even raise alerts under certain circumstances (e.g., when detecting a large number of outliers).

The parallelization strategy was based on the iterations performed when a sample was selected (one core assigned to each sample). Each core calculated all the statistics for sliding windows belonging to the same sample (not parallelized), taking advantage of memory caching mechanisms. Otherwise, sending sliding windows to different processors would result in more calls to main memory, as pages in cache would fail.

The parallel implementation showed its utility, especially when using up to three cores, and it is a topic to explore for more solutions. The code was not scaling as well as it could, but this might have been due to the machine and operating system we used. We believe that the performance and scalability could improve with a Linux-based system, and it would be deployable in multiple environments.

### 5.3. Research Limitations

This research is subject to several limitations. The first one is related to the generalization of the results to other domains beyond smart cities, environmental monitoring, home automation or agriculture. We did not have access to datasets from domains such as manufacturing or personalized medicine, in which sensors are also widely used today. Therefore, we will look for open datasets and industrial partners that will be able to share a few datasets that could improve our study.

On the other hand, the study of the effect of parallelization in edge devices requires access to certain hardware in order to be totally accurate with respect to the results. Even if the results obtained are questionable, access to specific devices, such as the BullSequana Edge (https://atos.net/en/solutions/bullsequana-edge, accessed on 29 August 2021), would show the potential of applying parallelization when processing sensor data.

### 5.4. Conclusions

The processing of sensor data is not easy, and we have seen that it requires some preliminary analysis. The solution we propose can facilitate understanding of the data, in line with widely applied methodologies such as CRISP-DM. Its added value is in the combination of several techniques based on statistics that provide valuable and crucial information about the data in early stages (even proposing a new way to interpret the outcomes from certain homogeneity tests), in a fast and efficient way (thanks to parallelization), and applicable to several domains without the need to (re-)train models.

Still there is room for improvement. The data from more sensor types will be analyzed to investigate the behavior of as many sensors as possible. Other aspects will be explored, including more data distributions and different transformations of the data, as well as new ways to improve and complement the detection of outliers.

Additionally, a deep analysis of the different properties of the data could be used to propose a mechanism that is able to perform sensor profiling, classifying and identifying them automatically.

Finally, we will continue exploring the parallelization of implementations, as it will gain more relevance in the near future and might be especially relevant if devices such as massively parallel processor arrays (MPPAs) become part of the mainstream environment, because they allow for much more parallelization at the core level.

## Figures and Tables

**Table 1:** Table comparing the outcomes of correlation tests facing different failures in the dataset.

Type of Failure/Test	Pearson	Kendall	Spearman
No error	0.916444	0.8414264	0.9399103
Malfunction	0.816932	0.752025	0.8621708
Bias	0.5387241	0.6189237	0.6764626
Drift	0.3560831	0.7168082	0.8339586

**Table 2:** Results of the performance analysis for the R parallelized scripts.

R Script	Results	Serial	2 Cores	3 Cores	4 Cores	5 Cores	6 Cores	7 Cores
Variation Process	Mean	94.72	48.42	34.37	27.73	34.77	23.58	31.99
	Best	57.48	45.23	24.05	21.39	29.52	21.08	26.87
	Worst	138.88	52.53	38.32	34.12	47.17	26.13	34.84
	C. of Variation	0.28	0.04	0.13	0.15	0.12	0.06	0.06
Outlier Process	Mean	471.42	247.58	212.99	208.1	154.95	160.91	186.21
	Best	432.64	235.89	199.65	178.57	143.91	144.05	169.06
	Worst	535.75	255.68	218.5	230.13	159.91	178.54	202.19
	C. of Variation	0.08	0.02	0.02	0.09	0.03	0.06	0.04
Correlation Process	Mean	4.36	2.82	2.47	2.45	2.63	2.75	2.83
	Best	4.09	2.68	2.36	2.39	2.55	2.53	2.77
	Worst	4.69	3.25	2.89	2.53	2.75	2.97	2.92
	C. of Variation	0.05	0.07	0.05	0.01	0.01	0.04	0.01

## Data Availability

Many of the datasets used, as well as the R scripts implemented are available in a GitHub repository (https://github.com/fjaviernieto/Sensor-Trust-Tools, accessed on 1 September 2021). That repository also includes data generated during our research.
